# Equipping Physical Activity Leaders to Facilitate Behaviour Change: An Overview, Call to Action, and Roadmap for Future Research

**DOI:** 10.1186/s40798-022-00423-0

**Published:** 2022-03-04

**Authors:** Mark Stevens, Tim Rees, Tegan Cruwys, Lisa Olive

**Affiliations:** 1 Research School of Psychology, The Australian National University, Canberra, ACT 2601 Australia; 2 Department of Rehabilitation and Sport Sciences, Bournemouth University, Poole, BH12 5BB UK; 3 School of Psychology, Deakin University, Geelong, VIC 3220 Australia; 4 IMPACT Institute for Mental and Physical Health and Clinical Translation, Deakin University, Geelong, VIC 3220 Australia

**Keywords:** Leadership, Exercise, Participation, Translation, Health behaviour change

## Abstract

Addressing high and stagnant physical inactivity rates remains a pervasive challenge for researchers, and a priority for health organisations, governments, and physical activity practitioners. Leaders are a prevailing feature of numerous physical activity contexts and can fundamentally influence people’s physical activity behaviours and experiences. In line with this, fitness companies and organisations commonly claim that the leaders of their classes, groups, or sessions will motivate, inspire, and ensure exercisers achieve their goals. We argue, however, that there is insufficient evidence regarding how leaders can best facilitate positive behaviours among, and outcomes for, group members to be confident that these claims are translating into strong physical activity leadership on the ground. In this article, we therefore call for research that equips leaders with greater knowledge and practical guidelines for how to maximise their effectiveness. To facilitate such research, we provide an overview of research that has examined the most effective ways for physical activity leaders to promote health-enhancing behaviours (e.g. greater participation) and positive experiences that may lead to such behaviours (e.g. greater exercise enjoyment) among those they lead. Then, with the shortcomings of this extant research in mind, we outline four broad recommendations for future research: (a) conduct research in novel and varied contexts, (b) focus on insufficiently active populations, (c) utilise qualitative methods, and (d) focus on translation and implementation. Such research would, we believe, generate knowledge that enables physical activity leaders to capitalise on their potential to be powerful agents of behaviour change.

## Key Points

- Physical activity leaders are a prevailing feature of many physical activity contexts.
- We summarise extant research examining how physical activity leaders can maximise their effectiveness, noting the limitations of this evidence base and arguing that it has provided insufficient practical guidance to facilitate strong physical activity leadership on the ground.
- To yield knowledge and guidance that equips leaders with the tools to act as powerful agents of behaviour change in the battle against physical inactivity, we propose that research should be conducted in novel and varied contexts, focus on insufficiently active populations, use qualitative methods, and focus on translation and implementation.

## Introduction

Physical inactivity is a substantial and widespread problem. Self-report data indicate that 27.5% of all adults worldwide are insufficiently active [1], while accelerometer data suggest that this figure may be as high as 90% [2]. The consequences of these high rates of inactivity are substantial. For example, it is estimated that, worldwide, approximately 7.2% of all deaths are attributable to physical inactivity [3], while a comprehensive analysis of data from 2013 estimated that the direct healthcare costs of physical inactivity exceeded US$53 billion that year [4]. Moreover, despite ambitious global targets to improve participation rates [5], and considerable efforts from researchers, practitioners, and policy makers, there is little to no evidence that physical activity rates are improving [1]. Understanding effective ways to promote and sustain people’s regular physical activity therefore remains a persistent challenge for researchers and practitioners.

Along these lines, it is clear that physical activity and its determinants do not exist in a social vacuum. Rather, people’s behaviours can be, and often are, influenced by others, including their friends, family members, social groups, and, of particular relevance to the present article, those with responsibility for guiding, instructing, and often motivating them: physical activity *leaders* [6–8]. However, research examining the most effective ways for physical activity leaders (e.g. fitness instructors, sports coaches, walking group leaders) to promote health-enhancing behaviours (e.g. sustained physical activity participation) and positive experiences that may lead to such behaviours (e.g. physical activity enjoyment) has been limited [9] and, we argue, generated little practical guidance from which such leaders can (confidently) draw. As a result, leaders’ capacity to effectively support, drive, and sustain physical activity behaviour change has been restricted—a missed opportunity in the battle to address physical inactivity. Indeed, because leaders are a salient feature of many physical activity contexts (e.g. fitness classes, exercise and rehabilitation groups, sports teams, and online workout videos), greater knowledge of how they can maximise their effectiveness and harness their influence would lay the platform for considerable and widespread benefits. Along these lines, while the capacity for physical activity leaders to reach, and therefore influence, sections of the population that do not engage in and are not attracted to structured exercise may be limited (and such subpopulations are not the focus of this article), it is noteworthy that large amounts of people regularly cycle into such settings but fail to maintain a high frequency of participation in the medium to long-term (e.g. see [10]). By more effectively supporting those that cycle into exercise settings to do this, and by supporting all attendees to participate regularly, maximise the benefits they derive from each session (e.g. by successfully encouraging moderate to high effort), and lead active lives more broadly, leaders therefore have the capacity to make an important difference (see also [11]).

With these points in mind, we extend a ‘call to arms’ for high-quality research that seeks to enhance understanding of how leaders can (a) promote positive physical activity behaviours and experiences among people who participate in the settings in which they operate, and (b) support and encourage those people to engage in frequent physical activity in daily life. Our goal in this article is to help facilitate and guide such research. To this end, we first briefly summarise extant research that speaks to how physical activity leaders can improve their effectiveness, noting the limitations of this evidence base. We then attempt to provide a roadmap for future research by making a series of recommendations, and identifying specific avenues, for research. We focus specifically on research with adult populations and orient our recommendations towards research with adults. However, we note both (a) the importance of research seeking to identify ways for youth physical activity leaders to maximise their effectiveness (which could also draw from our recommendations, albeit with caveats), and (b) that while there are likely differences, there is also likely to be overlap in how adult and youth physical activity leaders can achieve this.

## Existing Research on How Physical Activity Leaders can Promote Positive Physical Activity Behaviours and Experiences

### Early Research

Early research on physical activity leaders was characterised by a strong focus on the ‘style’ that leaders adopt, the behaviours they engage in, and their (perceived) competence, and how these factors influence how effectively they can promote positive group member behaviours and outcomes within the physical activity setting of interest. For example, researchers examined the benefits (e.g. for participants’ exercise class attendance and enjoyment) associated with group members’ (a) satisfaction with their leader’s enthusiasm, availability, motivation, and instruction [12, 13], (b) perception that their leaders are committed to providing a quality ‘service’, and are effective at interacting interpersonally and during tasks [14], and (c) perception that leaders are enthusiastic, able to communicate instructions, knowledgeable about fitness, and fit themselves [15]. However, with observational designs commonly used and the same factors rarely tested across multiple studies, this research failed to provide strong evidence that any of these factors distinguish effective from ineffective leaders.

The most popular focus of this work was examining the benefits of leaders engaging in an ‘enriched’ rather than ‘bland’ style. An enriched leadership style involves 'a high frequency of technical instruction, specific technical support, and positive skill-related feedback' [16, p. 123]. Conversely, a bland style involves leaders omitting support and encouragement, providing vague feedback, and focusing on negative corrections. Experimental studies demonstrated the superiority of an enriched compared to bland style for facilitating enjoyment of exercise [17, 18], self-efficacy and revitalisation following a one-off exercise session [16], and reduced social anxiety during exercise classes [19]. At a surface level, these findings appear promising; they indicate the potential for leaders to positively impact various important outcomes by engaging in a relatively simple set of behaviours. However, the ‘bland’ comparison group is problematic at best: behaviours such as giving vague feedback and focusing on negative corrections are arguably analogous to ‘bad’ leadership. It is therefore perhaps unsurprising that this style compared poorly to ‘enriched’ leadership. A ‘leadership as usual’ control condition would afford greater insight into the benefits of ‘enriched’ leadership.

More generally, efforts to identify specific behaviours, styles, or characteristics that set effective physical activity leaders apart largely lost traction in the early 2000s. These were replaced by two theory-driven streams of research, grounded in (a) self-determination theory [20–22] and (b) social identity leadership theory [23].

### Research Informed by Self-determination Theory

Self-determination theory was developed to help explain people’s motivations to engage in specific behaviours. Applied to physical activity leadership, at the most basic level the theory proposes that when, and to the extent that, leaders engage in behaviours that support people to feel autonomous, competent, and related (i.e. connected) to others (e.g. in their physical activity groups), this facilitates adaptive forms of motivation, and thus positive behaviours, experiences, and outcomes [24].

Empirical research has provided some support for this notion. Most notably, positive changes in key outcomes have been reported following interventions that trained exercise class leaders to use strategies that help fulfil class members’ needs for autonomy, competence, and relatedness (e.g. providing a meaningful rationale for activities, giving group members opportunities to make choices [25]). Specifically, participants in high (compared to low) ‘need supportive’ conditions have demonstrated greater class attendance, positive affect, and overall levels of physical activity post-intervention [26, 27], while within-group analyses have found increases in participants’ intentions to remain in classes across intervention periods [24]. There is also some evidence, primarily from observational studies, that when other types of leaders—including sports team coaches [28], walking group leaders [29], and physical activity counsellors [30]—engage in need supportive behaviours, group members experience similar benefits.

Although these findings appear promising, several limitations should be noted. Two of the three intervention studies conducted in exercise class contexts [26, 27] included small samples (*N* = 35 and *N* = 56); in the third, high attrition in the control condition forced the researchers to concentrate primarily on within-group effects [24]. Short or no post-intervention follow-ups and sub-optimal research designs (i.e. not randomised controlled trials) and control groups (i.e. not active dose-controlled) have also been consistent weaknesses of research to date. Notably too, these interventions had mixed or no effects on other outcomes, including vitality, negative affect, and, importantly, participants’ psychological need satisfaction (the theorised mechanism). This suggests that intervention refinement is needed to better align it with self-determination theory and thus enable stronger tests of the theory and its utility for improving exercise class leaders’ effectiveness (and physical activity leaders’ effectiveness more broadly).

### Research Informed by Social Identity Leadership Theory

The social identity approach states that categorising oneself in terms of a shared group membership (i.e. a social identity) underpins group behaviours, including social influence [31]. Social identity leadership theory builds on this. In short, it proposes that leadership is a process of social influence and that any leader’s capacity to exert influence (e.g. persuade people to increase their physical activity), as well as their effectiveness more generally, is greater to the extent that they (a) *create* a strong sense of shared identity in the group they are leading, (b) *represent* the group’s identity, (c) *advance* the group’s identity and interests, and (d) help *embed* the group’s identity in reality by providing practical activities that allow members to ‘live out’ their shared identity [23].

Growing evidence indicates that identity leadership is associated with favourable group member behaviours and experiences (e.g. greater performance and reduced burnout) in various contexts (e.g. organisational and political [32–34]). In the context of physical activity specifically, recent cross-sectional and two time-point survey studies have found promising evidence that, to the extent that group members perceive their leaders to engage in identity leadership, group members tend to participate in group sessions more frequently [35–37], exert more effort during sessions [37], and report that they are more likely to participate in future sessions [38]. However, these studies’ reliance on (a) non-experimental designs, and (b) subjective self-report measures to assess key outcome variables (e.g. participation and effort) mean further research is needed to more rigorously test the value of identity leadership (see also [39]).

## Advancing the Field: Foci for Future Research

As the preceding summary attests, the body of research examining how physical activity leaders can promote positive physical activity behaviours and experiences among exercisers remains small. This, combined with the shortcomings of most extant studies, limits the extent to which physical activity leaders—and those responsible for training them—can derive practical guidance to maximise their effectiveness. In the sections that follow, we seek to facilitate efforts to address this problem by outlining four broad recommendations for future research. A summary of these recommendations, and of some of the more specific avenues for future research we advocate, is provided in Table 1.

**Table 1:** Recommendations and specific avenues for physical activity leadership research

Recommendation	Avenues for research
1. Conduct research in novel and varied contexts	– Focus on underexplored contexts where leaders are present (e.g. video-guided workouts)
	– Examine context-specific differences in the optimal behaviours and strategies for leaders to adopt
	– Compare individual versus group settings; groups with consistent versus interchangeable members; settings with face-to-face versus virtual leaders
2. Focus on insufficiently active populations	– Screen for, and recruit, samples who are insufficiently active or who have recently begun engaging in structured exercise
	– Examine the effectiveness of different leadership styles and strategies for sustaining new exercisers’ participation
	– Aim to inform the development of materials that are specifically tailored for insufficiently active populations
3. Utilise qualitative methods	– Explore what people believe it is important for physical activity leaders to do, what makes them effective, and what this looks like in practice
	– Map findings against leadership theories to identify those with the greatest potential in the physical activity domain
	– Use qualitative methods to identify specific considerations for leaders working with insufficiently active populations
4. Focus on translation and implementation	– Identify concrete ways for leaders to engage in effective forms of leadership in practice
	– Develop interventions that aim to increase leaders’ capacity to facilitate people’s more frequent attendance of leader-led sessions, improved behaviours and experiences within sessions, and greater overall physical activity
	– Explore the feasibility and acceptability of such interventions for leaders, and barriers to their systematic uptake by those who would deliver them, with a view to maximising the potential for interventions to be widely and cost-effectively distributed

Importantly, the usefulness of *all* future research will, at least partly, hinge on its methodological rigour. Several detailed discussions of relevant considerations are available. For example, Antonakis et al. [40] outline the importance of testing causality and identify approaches that leadership researchers can use to achieve this in situations where randomisation (the gold standard) is not possible. Several articles have also addressed the issue of statistical power, with stricter criteria for appropriate sample sizes and more transparent reporting increasingly encouraged (e.g. [41, 42]). We will not elaborate on these points in detail here, but echo calls for leadership researchers to set high methodological standards [39, 43, 44].

### Recommendation 1: Conduct Research in Novel and Varied Contexts

First, research is needed that captures the full range of contexts in which physical activity leaders operate. Most research to date has been conducted in exercise classes. This remains a context in which many people engage in physical activity (and thus one which future research should not overlook). However, research in other contexts, and interrogating possible context-specific differences in the optimal ways for physical activity leaders to facilitate positive outcomes, is also sorely needed. This is because there may not be a single effective leadership approach that applies to all contexts and scenarios. It is possible, for example, that there may be differences in the optimal behaviours and strategies for leaders to adopt depending on whether they are in contexts where (a) people exercise individually or in groups, and (b) group members are consistent (e.g. running groups, sports teams) or interchangeable (e.g. drop-in exercise classes). This latter point would seem particularly pertinent for researchers adopting perspectives—such as the identity leadership approach—that place the group at the heart of their analysis.

It is also notable that researchers have neglected contexts in which physical activity leaders appear virtually in people’s homes through their screens and devices. Although the video-guided workout is a longstanding feature of the exercise industry, online platforms (e.g. YouTube) and the growing number of fitness companies (e.g. Les Mills) that offer workouts online as part of their business, mean that workout videos are now more readily accessible and popular than ever. *Peloton* is one company that has sought to capitalise on technological advances and people’s increasing desire (and at times need, particularly in the context of COVID-19) to exercise at home by centring their business around recorded and live-streamed exercise classes. However, while *Peloton* claims that their exercise classes feature ‘instructors that motivate’ [45], there is no empirical evidence pertaining to (a) how ‘virtual’ leaders in online settings can best motivate exercisers, or (b) whether or how the strategies and behaviours that these leaders deploy should differ from those of leaders in ‘real world’ (face-to-face) settings. Given the increasing popularity of exercise modalities involving virtual leaders, there is a clear need for research focused on understanding how such leaders can maximise their effectiveness.

### Recommendation 2: Focus on Insufficiently Active Populations

Second, research is needed that helps understand how to provide effective leadership for populations for whom physical activity behaviour change would confer the greatest benefit: insufficiently active individuals. Although more activity is generally better, the dose–response relationship between physical activity and its benefits is nonlinear; those who are least active benefit most from increasing their physical activity [46–48]. We therefore encourage efforts to uncover ways for leaders to both effectively encourage less active individuals to participate more frequently and with greater commitment in their own sessions, and to encourage such individuals to be more active more generally (i.e. in daily life).

Because studies focusing on physical activity leaders have typically used convenience samples of exercise class members, they have often not recruited participants who are among the least active in the population. As alluded to in the Introduction, however, it is common for less active people to cycle into these settings. The key challenge for these individuals tends to be sustaining a high frequency of participation in the medium- to long-term [10]. It is therefore important that future research in these settings attempts to specifically recruit new exercisers and examines the effectiveness of different leadership styles and strategies for helping them avoid the participation drop-off they often experience.

Separately, and following on from Recommendation 1, research in the context of workout videos would be well-suited to enabling researchers to specifically recruit insufficiently active participants. For example, researchers could administer screening questionnaires which require people to report their physical activity, and only include those whose physical activity falls below a threshold (e.g. public health guidelines). Subsequent experiments could involve participants watching, or conducting workouts led via, different pre-recorded workout videos where the leader’s behaviours have been manipulated. Participants could then rate the leader (e.g. on how engaging and motivational they are) and their own inclination towards doing the workouts. In instances where participants complete the workouts, measures of their effort, engagement, and experiences could be obtained through observation, self-reports, and wearable devices. Studies of this nature may not attract participants from certain backgrounds who may be less likely to participate in structured exercise (e.g. socially disadvantaged individuals), or those who simply have no interest in undertaking structured exercise. Nevertheless, such research could facilitate the development of exercise videos specifically tailored for low-active individuals who may be most willing to engage with them in the future (and for whom making them more appealing would thus have particular benefit) because these are the people who are most likely to choose to participate in the research.

### Recommendation 3: Utilise Qualitative Methods

To date, almost all physical activity leadership research has used a quantitative approach. We argue that qualitative research could yield novel and valuable insights and play a key role in generating applied knowledge that can inform leaders’ practice.

First, interviews or focus groups could be used as part of fundamental ‘back to basics’ research aiming to understand what people believe it is important for physical activity leaders to do, what makes them effective, and what this looks like in practice. Estabrooks et al. [49] made an initial attempt to examine some of these questions through semi-structured interviews. Specifically, these researchers explored older adults’ perceptions of the key characteristics for physical activity group leaders (of various forms) to possess. They found that their sample strongly valued leaders’ qualifications and competence, and ability to develop a bond with group members and create a positive physical and social environment (e.g. by using appropriate music and facilitating group integration). To develop understanding, future studies along these lines could examine whether themes that emerge from questions about what leaders should do, and what makes them effective, map against the key tenets of one or more of the numerous extant leadership theories [50]. The knowledge derived from such studies would support researchers to develop research grounded in the theories that show the greatest potential in this domain, with downstream benefits for the leadership evidence base upon which leaders can draw.

Qualitative methods could also be used to explore how to optimise the leadership of less active populations, and whether and how leaders should adapt their approach when working with these populations. For example, interviews or focus groups could be used to identify leadership factors that would (a) make less active people more likely to want to engage in exercise under their guidance or instruction and (b) deter them from wanting to do so. It is possible, for example, that there are aspects of leaders’ behaviours (e.g. using technical or ‘macho’ language) that contribute to inactive people feeling like they do not belong in certain exercise environments. Identifying such behaviours would facilitate practical guidance for leaders on the ground.

### Recommendation 4: Focus on Translation and Implementation

Finally, to truly unlock physical activity leaders’ potential to facilitate widespread behaviour change, researchers must concentrate not only on testing which forms of leadership are effective in the abstract, but also on (a) establishing concrete ways for leaders to engage in those forms of leadership in practice, and (b) developing effective interventions that enhance their capacity to do this. In this regard, progress to date has been restricted by the common use of survey instruments to assess leadership dimensions that assess these at the abstract level. This is particularly true for identity leadership research, which has heavily relied on the identity leadership inventory (ILI [51]) to assess the four identity leadership dimensions and (thus) their relationship with focal outcomes (e.g. [35–38]; see "Research Informed by Social Identity Leadership Theory" section above). The ILI items (e.g. ‘this leader acts as a champion for the group’) are well-aligned with identity leadership theory. However, at present, leaders on the ground may struggle to apply knowledge that acting as a champion for the group is beneficial, since research has not provided insights into *how* they can do this.

Although oriented towards sports performance, recent research has tested the effects of manipulating observable behaviours in line with the theory [52], and the efficacy of interventions seeking to develop sporting leaders’ identity leadership [53–56]. These are promising steps forward, particularly because some of the strategies used to enhance sports team members’ perceptions of their leader’s identity leadership in this research demonstrate potential applicability to other physical activity settings where health outcomes are typically a more central focus. For example, exercise group leaders could seek to use collective language, instigate the development of group norms and goals, and promote practical mechanisms (e.g. a WhatsApp group) through which people can ‘live out’ their social identity as an exercise group member. Researchers in sports contexts have also attempted to develop interventions informed by transformational leadership theory [57–59] and a system for coding transformational leadership behaviours—a tool with the potential to help leaders better understand what such behaviours ‘look like’ [60]. Again, some of the behaviours included in this tool appear transferable to other physical activity settings. For example, expressing confidence in what group members can achieve, highlighting the value and meaning of specific activities, and recognising and adapting to individual group members’ needs and abilities are all behaviours exercise group or class leaders could strive to deploy.

Nevertheless, much more research is needed (a) that leaders on the ground can readily use to guide how they behave, and (b) that focuses on developing interventions that help leaders behave more effectively. Indeed, only a handful of interventions have been developed and tested to enhance physical activity leaders’ capacity to improve people’s exercise behaviours and experiences with the goal of facilitating downstream health benefits [24, 26, 27]. Notably too, only one of these studies assessed the effect of the intervention on participants’ physical activity behaviours outside the exercise intervention setting (doing so over a relatively short five-week follow-up [26]). Further interventions are sorely needed that seek to develop physical activity leaders’ capacity to facilitate people’s more frequent attendance of sessions, improve their physical activity behaviours and experiences within sessions, and increase their overall physical activity levels. Although it may not be appropriate or realistic (particularly if resources are limited) for all interventions to aim for this latter goal [11], one tool intervention designers could draw on that may aid leaders’ efforts to improve people’s behaviours outside their own sessions is technology. For example, leaders could be encouraged to share videos providing encouragement or instruction, or to use social media (e.g. Facebook groups) to maintain communication with their group or class members outside sessions and encourage members to support each other [11, 61].

Finally, alongside these interventions, attempts to understand (a) their feasibility and acceptability for leaders (see [62]), and (b) barriers to their systematic uptake by those who would deliver them (i.e. in line with the goals of implementation science [63–65]), should also be undertaken. Ultimately, the objective should be to create interventions and programs that can be widely and cost-effectively distributed—for example as standardised (and potentially manualised) modules that exercise instructors receive as part of their training en route to accreditation.

## Conclusion

Leaders are a prevailing feature of numerous physical activity contexts and have the potential to play a substantial and valuable role in facilitating people’s greater engagement in, and more positive experiences of, physical activity. However, research to date has failed to provide sufficient knowledge or guidance to equip leaders with the tools to maximise their influence. Research addressing this and developing ways of translating this knowledge to leaders would enable them to act as agents of behaviour change in the battle against physical inactivity. We hope this article provides a stimulus and guide for this important research.

## Data Availability

Not applicable.
